# Goal-Directed vs Traditional Approach to Intraoperative Fluid Therapy during Open Major Bowel Surgery: Is There a Difference?

**DOI:** 10.1155/2019/3408940

**Published:** 2019-11-29

**Authors:** Prabhu P. Sujatha, Anitha Nileshwar, H. M. Krishna, S. S. Prasad, Manjunath Prabhu, Shobha U. Kamath

**Affiliations:** ^1^Department of Physiology, Melaka Manipal Medical College (Manipal Campus), Manipal Academy of Higher Education, Manipal, Karnataka, India; ^2^Department of Anaesthesiology, Kasturba Medical College, Manipal Academy of Higher Education, Manipal, Karnataka, India; ^3^Department of Surgery, Kasturba Medical College, Manipal Academy of Higher Education, Manipal, Karnataka, India; ^4^Department of Biochemistry, Kasturba Medical College, Manipal Academy of Higher Education, Manipal 576104, Karnataka, India

## Abstract

**Introduction:**

Optimum perioperative fluid therapy is important to improve the outcome of the surgical patient. This study prospectively compared goal-directed intraoperative fluid therapy with traditional fluid therapy in general surgical patients undergoing open major bowel surgery.

**Methodology:**

Patients between 20 and 70 years of age, either gender, ASA I and II, and scheduled for elective open major bowel surgery were included in the study. Patients who underwent laparoscopic and other surgeries were excluded. After routine induction of general anaesthesia, the patients were randomised to either the control group (traditional fluid therapy), the FloTrac group (based on stroke volume variation), or the PVI group (based on pleth variability index). Fluid input and output, recovery characteristics, and complications were noted.

**Results:**

306 patients, with 102 in each group, were enrolled. Five patients (control (1), FloTrac (2), and PVI (2)) were inoperable and were excluded. Demographic data, ASA PS, anaesthetic technique, duration of surgery, and surgical procedures were comparable. The control group received significantly more crystalloids (3200 ml) than the FloTrac (2000 ml) and PVI groups (1875 ml), whereas infusion of colloids was higher in the FloTrac (400–700 ml) and PVI (200–500 ml) groups than in the control group (0–500 ml). The control group had significantly positive net fluid balance intraoperatively (2500 ml, 9 ml/kg/h) compared to the FloTrac (1515 ml, 5.4 ml/kg/h) and PVI (1420 ml, 6 ml/kg/h) groups. Days to ICU stay, HDU stay, return of bowel movement, oral intake, morbidity, duration of hospital stay, and survival rate were comparable. The total number of complications was not different between the three groups. Anastomotic leaks occurred more often in the Control group than in the others, but the numbers were small.

**Conclusions:**

Use of goal-directed fluid management, either with FloTrac or pleth variability index results in a lower volume infusion and lower net fluid balance. However, the complication rate is similar to that of traditional fluid therapy. This trial is registered with CTRI/2018/04/013016.

## 1. Introduction

Fluid therapy is an integral part of care of patients undergoing major abdominal surgery. Accurate assessment of fluid status of a patient is an important goal in the operation theatre for the anaesthetist. It is important to optimize the haemodynamics perioperatively to improve outcome of the patient and reduce mortality [1]. The fact that inadequate fluid replacement can lead to inadequate tissue perfusion and prerenal failure is well known. However, it is believed that a little excess fluid will be tolerated and adjusted by otherwise healthy patients with normal kidneys. It is not well recognised that hypervolaemia can exacerbate loss of glycocalyx on the capillary endothelium (double barrier) with consequent interstitial edema [2–4] and possibly intestinal anastomotic leaks.

There has been a lot of controversy in the recent literature as to what constitutes “optimal”. There are references to liberal, standard, and restrictive fluid regimens, but their definitions are still unclear [5] and no consensus has been reached as to what strategy must be used. An average anaesthetist is still confused as to whether third space loss must be accounted for at all, or if considered, how much should it be during these surgeries [6, 7]. A method that is objective and accurate would help eliminate guesswork involved in fluid therapy currently in these situations. There is increasing evidence in the literature advocating the use of individualised goal-directed fluid therapy guided by dynamic indicators of fluid responsiveness such as arterial pressure-based stroke volume variation, pulse pressure variation, and systolic pressure variation [8–11]. The pleth variability index can be obtained from a Masimo pulse oximeter and is totally noninvasive unlike the other parameters.

This study was undertaken to prospectively compare goal-directed intraoperative fluid therapy using FloTrac (Vigileo monitor) or pleth variability index (Masimo Radical-7 monitor) with traditional fluid therapy in general surgical patients undergoing open major bowel surgery.

## 2. Materials and Methods

This prospective study was conducted after obtaining approval from the Institutional Ethics Committee (Kasturba Hospital, Manipal. IEC: 463/2012). Patients aged between 20 and 70 years, of either gender, belonging to ASA I and II physical status, and scheduled for elective open major bowel surgery such as on the small intestine, colon, or stomach requiring invasive arterial pressure monitoring, for e.g., colonic resection, intestinal resection, and anastomosis or gastrectomy were included in the study. Patients undergoing laparoscopic surgery, major vascular surgery, and surgery on the liver or urogenital system were excluded. Similarly, patients with a history of cardiac failure or renal failure were excluded. Patients who underwent laparotomy followed by simple colostomy or jejunostomy were also excluded.

The principal investigator assessed the patients on the day prior to surgery for suitability of their inclusion in the study and ensured that no exclusion criteria were present. Once enrolled, written informed consent was taken from all patients before the surgery. Bowel preparation was done as per the surgeon's instructions on the day prior to surgery. They were kept fasting as per standard “nil per oral” guidelines.

All patients were premedicated with tab alprazolam 0.25 mg, the night prior and on the morning of surgery. In the operating room, patent peripheral intravenous access (18 G or larger) was secured. General anaesthesia with endotracheal intubation was done after induction with 2–2.5 mg/kg of propofol, 2 *μ*g/kg of fentanyl, and neuromuscular blockade with vecuronium 0.1 mg/kg. Monitoring included pulse oximetry, noninvasive blood pressure and electrocardiogram (lead II and V5), capnography, anaesthetic agent analyser (to maintain a minimum alveolar concentration (MAC) of 1–1.3), urinary catheter, and nasopharyngeal temperature. Any additional monitoring such as central venous pressure was done at the discretion of the attending anaesthesiologist. Anaesthesia was maintained using isoflurane in a mixture of nitrous oxide and oxygen. All patients were ventilated with a tidal volume of 8 ml/kg and at a rate required to maintain normocarbia. Epidural analgesia was provided along with general anaesthesia unless difficult. In such cases, intravenous morphine was used. Analgesia and titration of the anaesthetic were done by the anaesthetist in charge of the patient.

The patients were randomised into one of the three groups: control group, FloTrac group, and PVI group, using computer-generated random numbers and sealed envelope technique by the second investigator. All patients underwent surgery in the supine position. The patients in the control group received intravenous fluids according to the current practice—2 ml/kg/h of surgery for maintenance and 6 ml/kg/h of actual surgery (from incision to skin closure). Additional boluses of crystalloid, colloid (hydroxyethyl starch), or blood products were based on the subjective assessment of blood loss at the discretion of the attending anaesthesiologist.

Patients in the FloTrac group had a radial arterial line (20 G) secured for continuous monitoring of arterial blood pressure after induction of anaesthesia. The FloTrac sensor was attached to the arterial line and connected to the Vigileo monitor—3^rd^ generation (Edwards Lifesciences, Irvine, California, USA). Once patient data such as age, sex, height, and weight were entered, the system computed stroke volume from the patient's arterial pressure signal and displayed cardiac index and SVV continuously. The baseline readings of stroke volume variation (SVV) were noted, and the patient was monitored continuously thereafter with FloTrac® in addition to standard monitoring.

Patients in the PVI group, had a pulse oximeter probe (LNCS; Masimo Corp.) placed on the index finger of one hand (contralateral to the side of the BP cuff) and connected to a Masimo Radical-7 monitor with PVI software (version 7). PVI calculation was accomplished by measuring changes in the perfusion index over a time interval sufficient to include one or more complete respiratory cycles according to the inbuilt algorithm. The lower the number, the lesser the variability in the PVI over a respiratory cycle. The baseline PVI was recorded in the PVI group and then monitored continuously thereafter with the Masimo Radical-7 monitor in addition to standard monitoring.

In all groups, the baseline fluid therapy was 2 ml/kg/h for maintenance. In the FloTrac group, additional fluid therapy was guided by FloTrac®. The baseline stroke volume variation was noted. 200 ml of hydroxyethyl starch (HES) was given over 10 min. If the stroke volume variation showed 13% or more, an additional bolus of 200 ml of HES was given over the next 10 min. If the stroke volume variation was less than 13%, no more bolus of fluid was given. The process was repeated until the stroke volume variation was within 13%.

In the PVI group, additional fluid therapy was guided by a Masimo pulse oximeter. The baseline readings of PVI were noted. 200 ml of HES was given over a period of 10 min. If PVI was >13%, an additional bolus of 200 ml HES was given over the next 10 min. If there was no increase in PVI or it was less than 13%, no more bolus of fluid was given. The process was repeated until the increase in PVI with the fluid therapy was within 13%. HES was given up to a maximum of 20 ml/kg as required beyond which fluid boluses were done using Ringer lactate in both the FloTrac and PVI groups.

In all groups, maximum allowable blood loss was calculated as follows [12]:

(1) $$\begin{array}{c} \frac{\text{Body weight}\times 70\left(\text{preoperative haemoglobin}\left(g\%\right)-\text{target haemoglobin}\left(g\%\right)\right)}{\text{average haemoglobin}\left(g\%\right)}, \end{array}$$

where average haemoglobin (*g*%) = [(preoperative Hb + target Hb)/2].

Allowable blood loss was replaced with colloids (hydroxyethyl starch) up to 20 ml/kg including the fluid boluses given during the procedure. Any blood loss exceeding the allowable blood loss was replaced with packed red cells. Fresh frozen plasma and platelets were transfused when massive blood transfusion was required.

In all the three groups, use of inotropes or vasopressors was done only after euvolaemia was ensured as assessed by the attending anaesthesiologist. If a vasopressor was required, small doses of mephentermine were given intermittently. Efforts to avoid intraoperative hypothermia included use of body warmer, fluid warmer, and heat and moisture exchanger in all patients.

All patients were monitored with blood pressure, heart rate, and electrocardiogram postoperatively for at least 24 h.

Every patient was followed up postoperatively by the principal investigator, and the following data were collected: Type and amount of fluids/blood products administered, urine output, and blood and fluid loss in drains postoperatively. The time to return of bowel movement and time to oral intake were noted. The requirement of ICU care including postoperative ventilator support, haemodynamic instability requiring vasopressor support, organ dysfunction, length of ICU stay (if applicable), and hospital stay were also recorded.

The primary outcome measure was postoperative morbidity. Morbidity in terms of complications was classified as per Dindo et al. [13] and given in Table 1.

Presence of wound infections, wound dehiscence, and secondary suturing and occurrence of anastomotic leaks were recorded. In addition, infection occurring elsewhere (urinary tract infection, pneumonia) was also noted. If any culture/sensitivity of any fluid/secretion were obtained, the results of such tests were noted.

Secondary outcome measures were rise in serum lactate levels as a measure of global perioperative circulatory inadequacy, return of bowel movement, oral intake, duration of ICU stay, HDU stay, time to readiness for discharge, hospital stay, and mortality.

The sample size was determined based on a pilot study which showed that the patients who developed complications had received more amounts of intraoperative fluids (11 ± 6 ml/kg/h vs 8 ± 3 ml/kg/h). For an alpha error of 0.05 and 90% power, 94 patients needed to be studied in each group. We enrolled 102 patients in each group.

Summarizing the data for demographic variables was done. Chi-square test was performed to find out the association between the categoric variables. Kruskal–Wallis test was performed for parameters with nonparametric distribution between three groups. If found significant, Mann–Whitney *U* test was done for pairwise comparison. Repeated measures of ANOVA were obtained for lactate values. One-way ANOVA was used for data with normal distribution.

## 3. Results

A total of 336 patients were assessed for eligibility. Among them 23 patients, refused to give consent and 7 cases were cancelled because patients were not willing to undergo surgery.

**Figure 1:**
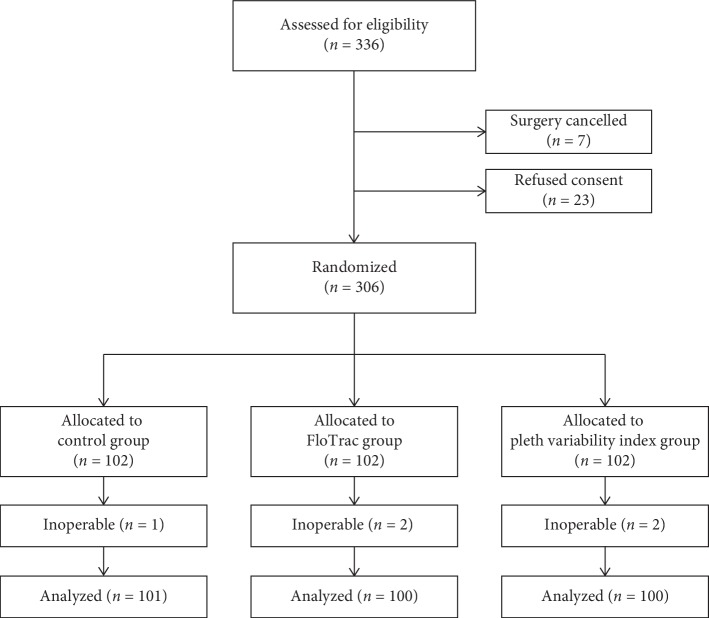
Consort diagram of the study.

Hence, a total of 306 patients who fulfilled the criteria were enrolled in the study. 102 patients were allocated to each group. Out of 102 patients, 1 patient in the control group, 2 patients in the FloTrac group, and 2 patients in the PVI group turned out to be inoperable (Figure 1).

The demographic data, ASA PS, and anaesthetic technique used are shown in Table 2. There was no statistically significant difference between the groups with respect to age, gender, height, weight, ASA physical status, and anaesthetic technique.

The duration of surgery and the various surgical procedures are given in Table 3. There was no statistically significant difference between three groups with respect to duration of surgery or the surgical procedures.

**Figure 2:**
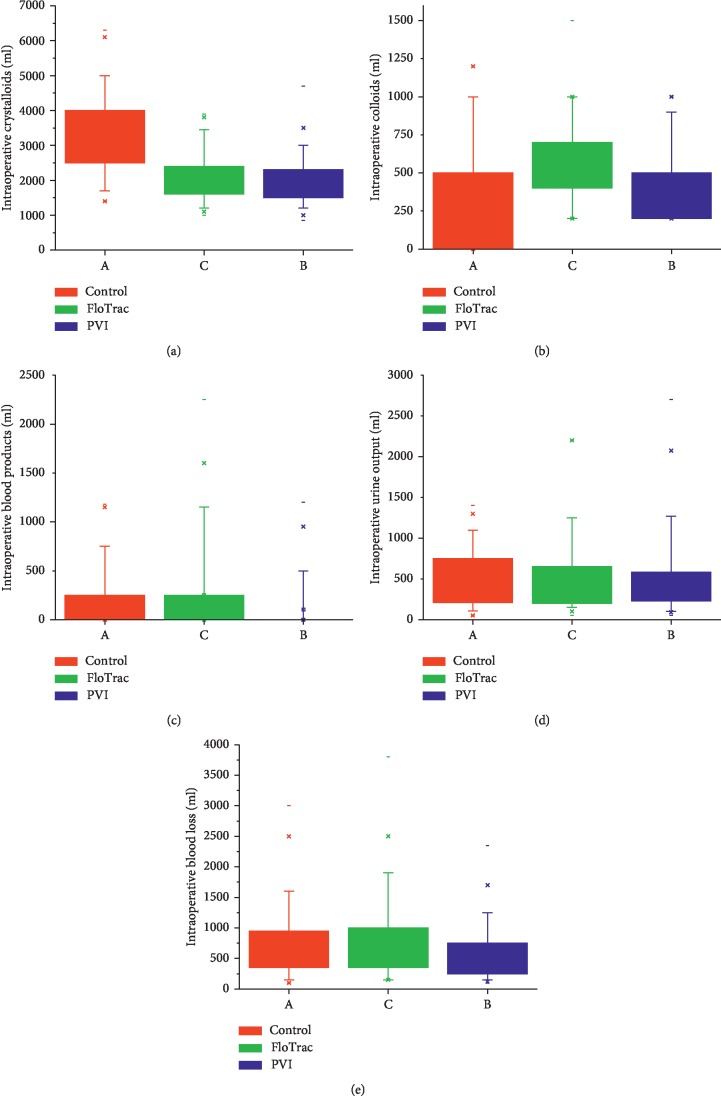
Comparison of intraoperative fluids (a, b), blood products (c), urine output (d), and blood loss (e) in the three groups. Data expressed in median (IQR). Kruskal–Wallis test used.

The amount of intraoperative fluids, blood products, blood loss, and urine output in the three groups are given in Figure 2. The control group received significantly more amounts of crystalloids and lesser amount of colloids as compared to the other two groups. FloTrac and PVI groups were similar with regard to fluid administration. Blood loss was significantly more in the FloTrac group as compared to the PVI group, but no difference was seen between the control and PVI groups. Intraoperative urine output was similar in all three groups.

**Figure 3:**
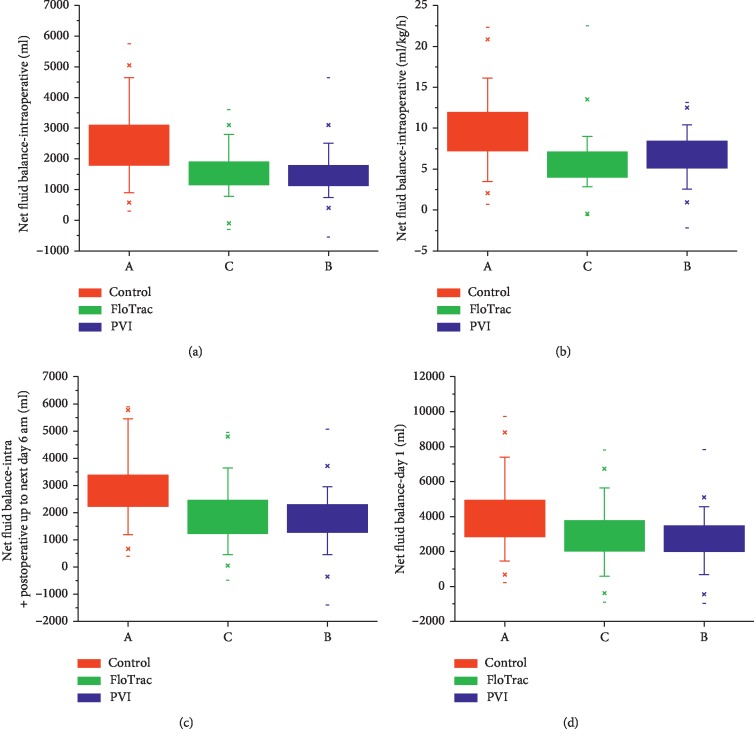
Net fluid balance (NFB) in ml (a) and ml/kg/h (b) for the intraoperative period and cumulative NFB for the postoperative period up to the next day 6 am (c) and postoperative day 1(d) (continued from the intraoperative period). Data expressed in median (IQR). Kruskal–Wallis test used.

The net fluid balance (NFB) calculated (as difference between input and output) for the intraoperative period showed that patients in the control group had a statistically and clinically significant positive fluid balance compared to the FloTrac and PVI groups (Figure 3). Similar results were seen for cumulative amount of fluids (including intraoperative and postoperative fluids) given up to the immediate postoperative period and for postoperative day 1. There was no difference between the FloTrac and PVI groups. The NFB was compared pairwise between the three groups using Mann–Whitney *U* test. There was a statistically significant difference between the control and FloTrac, and control and PVI groups (*p*=0.001), whereas no difference was found between the FloTrac and PVI groups.

**Figure 4:**
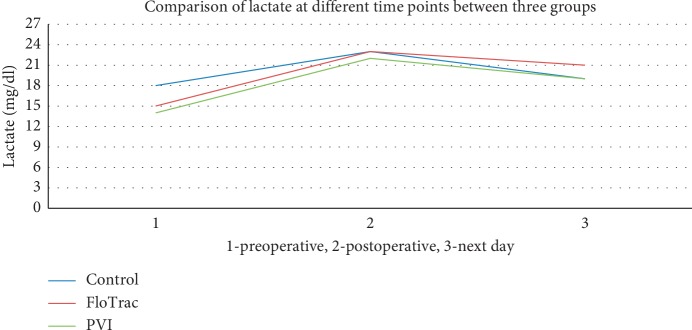
Change in serum lactate levels in the three groups.

Repeated measures of ANOVA were done to compare the lactate level at different time points between the groups and within the groups. There was a mild to moderate increase in serum lactate levels in all three groups. However, the average rise was up to 23 mg% and did not go beyond 44 mg% (levels considered significant lactic acidosis in shock) (Figure 4).

The days to ICU stay, HDU stay, return of bowel movement, days to oral intake, duration of hospital stay, and survival rate are given in Table 4. The groups were largely comparable in all the above parameters. However, statistically significant but clinically insignificant difference was shown for days to oral intake between the control group and the PVI group (*p*=0.046).

**Figure 5:**
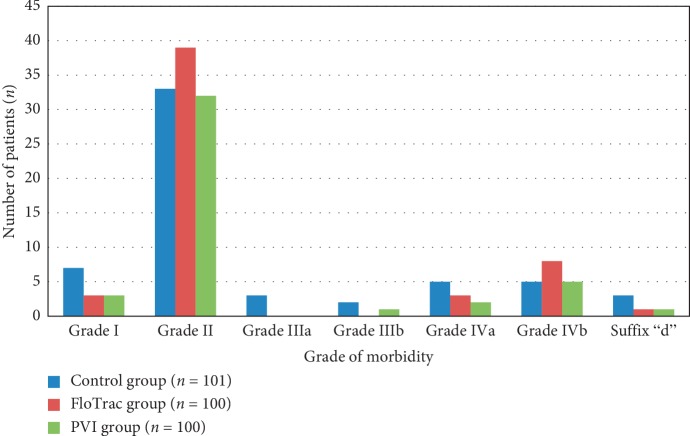
Postoperative morbidity.

Morbidity was graded based on severity of complications as suggested and validated by Dindo et al. [13]. The number of patients developing these complications in all the three groups is given in Figure 5. No difference could be demonstrated between the three groups with respect to morbidity. In this graph, however, the highest grade of complication developed by patients has been shown.

Table 5 shows the number of patients developing anastomotic leak and renal dysfunction graded as per KDIGO guidelines [14]. Out of 11 patients in the control group who developed renal dysfunction, 3 recovered with conservative management. Eight patients required relaparotomy of whom 3 recovered. The remaining 5 patients developed multiorgan dysfunction and died. In the FloTrac group, only one patient had anastomotic leak, developed multiorgan dysfunction, and recovered after relaparotomy. In the PVI group, of the 4 patients who had anastomotic leak, 3 developed multiorgan dysfunction and died, while one patient recovered.

## 4. Discussion

Perioperative fluid therapy is one of the most debated areas in the present day, in anaesthetic practice. Assessment of the adequacy of the intravascular volume is of prime importance to avoid hypovolemia and tissue hypoperfusion [1]. The postoperative complications associated with major surgery have a huge impact on short-term and long-term mortality. The incidence of these complications could decrease the median survival by 69% [15, 16].

In this study, we included only major open abdominal surgeries such as Whipple's procedure, gastrectomy, abdominoperineal resection, hemicolectomy, low anterior resection, sigmoid colectomy, gastrojejunostomy, and jejunojejunostomy since these were expected to have large fluid shifts unlike laparoscopic surgeries. The demographic data, surgical procedures and their duration, and anaesthetic technique were all comparable between the groups.

We included only patients with ASA I and II to minimize confounding factors. Goal-directed fluid therapy, guided by SVV and PVI, has resulted in reduced fluid infusion in the present study. The control group had a net fluid balance of almost 2500 ml.

Both crystalloids and colloids can be used for resuscitation and volume replacement. Crystalloids were used for maintenance, and colloid used for fluid challenges. Albumin is available as 20% and is expensive. Gelatin is available as Haemaccel with a high incidence of anaphylactic reactions. Hence, we used 6% hydroxyethyl starch as the colloid for intervention in both the FloTrac group and the Masimo group. Colloid was given as required up to a maximum of 20 ml/kg beyond which fluid boluses were given using Ringer lactate.

Comparison of administration of crystalloids and colloids between the groups showed that the control group had received significantly more amount of crystalloids, median (IQR) (3253, 2450–4000 ml) compared to the FloTrac group (2000 ml, 1600–2437 ml) and the PVI group (1875, 1500–2300 ml). Intraoperative colloids were significantly more in the FloTrac and PVI group compared to the control group. This is very similar to other studies in the literature [17–21]. There was a nonsignificant trend showing a decreased fluid requirement for the first 24 h postoperatively in patients receiving goal-directed fluid therapy, similar to other studies [22–25]. These studies also mention that the end-surgery fluid balance was significantly lower with goal-directed fluid management compared to conventional fluid management.

We calculated the NFB by adding up all fluid infused (crystalloids, colloids, and blood products) and subtracting all measurable fluid losses (blood loss and urine output). The NFB calculated for the intraoperative period showed statistically and clinically significant positive fluid balance in the control group (median of 2500 ml; 9 ml/kg/h) compared to the intervention group (FloTrac: median of 1515 ml; 5.4 ml/kg/h and PVI group: median of 1420 ml; 6 ml/kg/h). Similar results were seen when the cumulative NFB was calculated for the immediate postoperative period up to the next day 6 am (includes intraoperative and postoperative fluids) and for postoperative day 1 and 2 for cumulative amount of fluids.

Increased extracellular fluid in the bowel can lead to decreased gastrointestinal motility, gastrointestinal edema, and possibly ileus [26]. Intestinal edema can cause tension at bowel anastomoses and may contribute to anastomotic dehiscence [27]. Rarely, massive fluid restoration may be associated with acute ascites [28].

In colorectal surgery, administration of too much fluid perioperatively may cause pneumonia and respiratory failure, intestinal edema, renal diuresis, inhibit bowel movements, postoperative ileus, and delayed wound healing due to increased cutaneous edema [27].

Morbidity was graded based on severity of complications as suggested and validated by Dindo et al. [13]. Majority of the earlier studies showed goal-directed fluid management improves the patient's outcome (reduces postoperative complications, lactate level, length of hospital stay, ICU stay, days to oral intake, day of return of bowel movement, and mortality) [22–25, 29]. There was no difference between the three groups with respect to development of MODS or survival.

In our study, though the lactate levels increased significantly following surgery in all three groups, no statistically significant difference was seen between the groups at different time points (preoperative, immediate postoperative period, and the next day). The lactate levels in all the three groups were within the limit of lactic acidosis (23 mg/dl) despite long-duration surgery. Mortality was similar in all three groups.

Many earlier studies showed that GDFT improves patient outcome (reduces postoperative complications, lactate level, length of hospital day, ICU stay, days to oral intake, day of return of bowel movement, and mortality) [18, 21].

Ramsingh et al. [30] and Salzwedal et al. [31] observed that the total number of complications was significantly lower in the study group. Infectious complications were significantly reduced. There were no significant differences in the return of the first bowel movement after surgery. Scheeren et al. [32] in their study found that the proportion of patients with at least one complication and the number of postoperative complications per patient was lower in the GDT group. They also concluded that goal-directed strategy might decrease postoperative organ dysfunction. The present study showed no significant difference for day of return of bowel movement, toleration of diet, serious complication, and mortality rate.

A total of 20 (6.67%) patients in this study developed postoperative renal dysfunction (first 48 hours) (control group: 10, FloTrac group: 7, and PVI group: 3). Although the incidence of renal dysfunction seems higher in this study as compared to Grass et al. [33], 14 of the 20 patients had only Stage I renal dysfunction and recovered. There was no difference between the three groups.

This was a prospective, randomised controlled study, addressing the need for more close monitoring and goal-directed perioperative fluid therapy for patients undergoing major open abdominal surgery. It addressed the utility of FloTrac and pleth variability index to implement goal-directed therapy. The study suggests that anastomotic leaks are more frequent in patients who receive traditional fluid therapy. However, the numbers are too small to confirm the same.

It was a single-centred trial. The occurrence of postoperative complications is multifactorial and depends on the age, comorbidities, surgical expertise including use of staplers, and other intraoperative complications. These could not be standardized in all patients. The study was commenced in 2014, and the protocol was designed on what was applicable at the time. There was not much change in the management of patients till the end of the study (2018). Enhanced recovery program has since then been adopted. The relationship between fluid management and the development of anastomotic leak appears significant but will need to be examined in a much larger study.

## 5. Conclusions

The use of goal-directed fluid management using stroke volume variation obtained through the minimally invasive FloTrac Vigileo™ or using the pleth variability index from Masimo Radical-7 results in a lower net fluid balance as compared to traditional fluid therapy. The study suggests that the number of patients developing anastomotic leak is higher with traditional fluid therapy as compared to goal-directed management. However, the study is underpowered to confirm statistical significance of this complication alone. This will need to be evaluated further with a larger study. There is no evidence of any influence of fluid management on the length of hospital stay, time to readiness for discharge, stay in the intensive care unit postoperatively, days to oral intake, day of return of bowel movement, lactate levels, and mortality rate.

## Figures and Tables

**Table 1:** Grades of postoperative complications (as adapted from Dindo et al. [13]).

Grade 1: confusion, noninfectious diarrhea, transient elevation of creatinine
Grade 2: infectious diarrhea, blood transfusion, wound/urinary tract/blood/sputum infection
Grade IIIa: requiring surgical intervention not under GA (secondary suturing under local anaesthesia)
Grade IIIb: requiring surgical intervention under GA (anastomotic leak requiring general anaesthesia for repair)
Grade IVa: single organ dysfunction requiring ICU
Grade IVb: multiorgan dysfunction
Suffix “d”: if the patient suffers from a complication at the time of discharge, the suffix “d” (for disability) is added to the respective grade of complication. This label indicates the need for a follow-up to fully evaluate the complication.

**Table 2:** Demographic data.

Parameter	Control group (*n* = 101)	FloTrac group (*n* = 100)	Pleth variability index group (*n* = 100)	*p* value
Age (years) mean (SD)^*∗*^	52 (12)	53 (11)	53 (12)	0.744
Weight (kg) mean (SD)^*∗*^	54 (11)	56 (10)	52 (12)	0.091
Height (cm) mean (SD)^*∗*^	161 (8)	160 (9)	159 (8)	0.348

Gender^*∗∗*^*N* (%)				
Male	68 (67.3)	68 (68)	59 (59)	0.332
Female	33 (32.7)	32 (32)	41 (41)	

ASA physical status^*∗∗*^*N* (%)				
I	59 (58.4)	47 (47)	48 (48)	0.196
II	42 (41.6)	53 (53)	52 (52)	

Anaesthetic technique *N* (%)				
GA + epidural	95 (94.1)	93 (93)	88 (88)	0.240

^*∗*^ANOVA and ^*∗∗*^chi-square test; ASA = American Society of Anesthesiologists; GA = general anaesthesia.

**Table 3:** Comparison of duration of surgery and the surgical procedures in the three groups.

Parameter	Control group (*n* = 101)	FloTrac group (*n* = 100)	Pleth variability index group (*n* = 100)
Duration of surgery (min)^*∗*^(median ± interquartile range)	270 (210, 430)	275 (212, 390)	240 (210, 330)
Surgical procedure *n* (%)^*∗∗*^			
Whipple's procedure/triple bypass	29 (28.7)	29 (29)	20 (20)
Abdominoperineal resection/low anterior resection	15 (14.8)	19 (19)	12 (12)
Hemicolectomy/sigmoid colectomy/proctocolectomy	23 (22.7)	23 (23)	32 (32)
Total/distal gastrectomy	16 (15.8)	17 (17)	23 (23)
GIST (excision and intestinal anastomosis)	2 (1.9)	1 (1)	1 (1)
GJ + JJ	16 (15.8)	11 (11)	12 (12)

GIST = gastrointestinal stromal tumour; GJ + JJ = gastrojejunostomy + jejunojejunostomy; ^*∗*^*p*=0.312 Kruskal–Wallis test and ^*∗∗*^*p*=0.544 chi-square test.

**Table 4:** Comparison of ICU and HDU requirement, ICU and HDU stay, days to return of bowel movement, days to oral intake, days of hospital stay, and survival rate in the three groups. Values are presented as number (percentage) or as median (interquartile range).

Parameter	Control group (*n* = 101)	FloTrac group (*n* = 100)	Pleth variability index group (*n* = 100)	*p* value
ICU requirement *n* (%)	19 (18.8)	20 (20)	12 (12)	0.284
Length of ICU stay (days) median (IQR)	(**n** = **19**) 2 (1, 3)	(**n** = **20**) 2 (1, 5)	(**n** = **12**) 1 (1, 3)	0.296
HDU requirement *n* (%)	63 (62.4)	62 (62)	58 (58)	0.786
Length of HDU stay (days) median (IQR)	(**n** = **63**) 3^*∗*^ (2, 4)	(**n** = **62**) 2 (1, 3)	(**n** = **58**) 2^*∗*^ (1, 3)	0.004
Day of return of bowel movement (days) median (IQR)	3 (2, 4)	3 (2, 3)	2 (2, 3)	0.156
Days to oral intake (days) median (IQR)	3^*∗∗*^ (2, 4)	3 (2, 4)	4^*∗∗*^ (2, 5)	0.047
Length of hospital stay (days) median (IQR)	14 (11, 17)	14 (11, 17)	13 (11, 16)	0.427
Survival *n* (%)	93 (92.1)	92 (92)	94 (94)	0.887

ICU = intensive care unit; HDU = high dependency unit. ^*∗*^control vs PVI (0.004); ^*∗∗*^control vs PVI (0.047). Data were compared using chi-square test and nonparametric Kruskal–Wallis test.

**Table 5:** Patients who developed anastomotic leak and renal dysfunction in all the three groups.

	Control group (*n* = 101)	FloTrac group (*n* = 100)	PVI group (*n* = 100)	*p* value
Anastomotic leak (*n*)	11	1	4	0.006
Postoperative renal dysfunction in the first 48 hours (*n*)				
Stage I	9	3	2	0.261
Stage II	1	3	1	
Stage III	0	1	0	

Chi-square test.

## Data Availability

Data used to support the findings of this study are included within the article.
